# Mechanistic remodeling and immunoregulatory functions of the B cell–humoral immunity axis in inflammatory bowel disease

**DOI:** 10.3389/fimmu.2026.1857614

**Published:** 2026-07-09

**Authors:** Tao Zhang, Zhetan Ren, Hongkun Zhang, Zhengchao Pan, Meng Chen, Siyuan Bu, Xiaozhen Cheng, Jirun Peng, Yongduo Yu

**Affiliations:** 1 Liaoning University of Traditional Chinese Medicine, Shenyang, China; 2 Second Affiliated Hospital, Liaoning University of Traditional Chinese Medicine, Shenyang, China; 3 Department of General Surgery, Beijing Shijitan Hospital, Capital Medical University, Beijing, China; 4 Shenzhen Traditional Chinese Medicine Hospital, Shenzhen, China; 5 Ninth School of Clinical Medicine, Peking University, Beijing, China

**Keywords:** antibody repertoire, B cells, humoral immunity, inflammatory bowel disease, tertiary lymphoid structures

## Abstract

Inflammatory bowel disease (IBD) is an immune-mediated disorder characterized by chronic inflammation of the intestinal mucosa, arising from dysregulation across multiple layers of immune control. In recent years, the contribution of B cells and humoral immunity to IBD pathogenesis has gained increasing attention. B cells participate in mucosal immune regulation through differentiation, clonal expansion, and antibody production, and are closely associated with the formation and structural organization of local tertiary lymphoid structures (TLSs). Beyond antigen neutralization, antibodies can modulate inflammatory cell activation and tissue injury via Fc receptor signaling, complement activation, and immune complex–mediated responses. In parallel, intestinal barrier integrity and the extent of antigen exposure critically influence B cell activation and antibody output, forming an interconnected network of local immune processes. Therapeutic strategies targeting the B cell–humoral immunity axis are emerging, including B cell–directed therapies, modulation of antibody effector pathways, and restoration of mucosal immune function. Although accumulating evidence suggests that these approaches may confer benefits in controlling inflammation and modulating immune responses, their efficacy and applicability vary across different strategies and patient populations. In this review, we systematically integrate current evidence on B cell differentiation, local lymphoid organization, and humoral immune responses in IBD. We propose the “B cell–humoral immunity regulatory axis” as a conceptual framework to delineate mucosal immune remodeling in IBD, with the aim of advancing mechanistic understanding and informing the optimization of therapeutic interventions.

## 1 Introduction

Inflammatory bowel disease (IBD) comprises a group of non-infectious disorders characterized by chronic, relapsing intestinal inflammation, primarily including Crohn’s disease (CD) and ulcerative colitis (UC) (1, 2). Its pathogenesis is driven by the persistent interplay of multiple factors, including genetic susceptibility, epithelial barrier disruption, microbial dysbiosis, and dysregulated immune responses (3). In recent years, biologics and small-molecule targeted therapies have markedly reshaped the therapeutic landscape of IBD (4). However, in real-world settings, a substantial proportion of patients continue to experience primary or secondary non-response, persistent low-grade inflammation, and progressive tissue damage (5). These challenges indicate that current conceptual frameworks remain insufficient to fully explain sustained mucosal inflammation, local immune architectural remodeling, and disease heterogeneity in IBD.

Historically, immunological research in IBD has been largely centered on T cells and their associated inflammatory mediators, while the role of B cells and humoral immunity has received comparatively less emphasis (6). With the advent of single-cell sequencing, spatial transcriptomics, and B cell receptor (BCR) repertoire profiling, the contribution of B cells to IBD is being increasingly redefined. Emerging evidence suggests that, beyond antibody production, B cells participate in antigen presentation, maintenance of immune tolerance, and regulation of local inflammatory responses (7, 8). Concurrently, B cell–associated immune processes are intricately linked to the remodeling of mucosal lymphoid structures and persistent alterations in humoral effector responses, together shaping the evolving immune microenvironment of the intestinal mucosa in IBD (9). These advances underscore the need to expand the conventional T cell–centric paradigm toward a more integrated framework that incorporates B cells and humoral immunity.

In this context, the present review focuses on three interconnected dimensions: remodeling of B cell differentiation, spatial disorganization of TLSs, and alterations in antibody-mediated effector networks. We examine how these processes are coordinated within the same mucosal immune microenvironment and how they collectively influence inflammatory states and tissue responses in IBD. By systematically integrating current evidence on B cells, TLSs, and humoral immunity, this review aims to delineate their sequential and interrelated roles in disease pathogenesis, and to explore the potential implications of this framework for disease stratification and precision therapeutic strategies.

## 2 B Cells in the intestinal immune microenvironment of IBD

**Figure 1:**
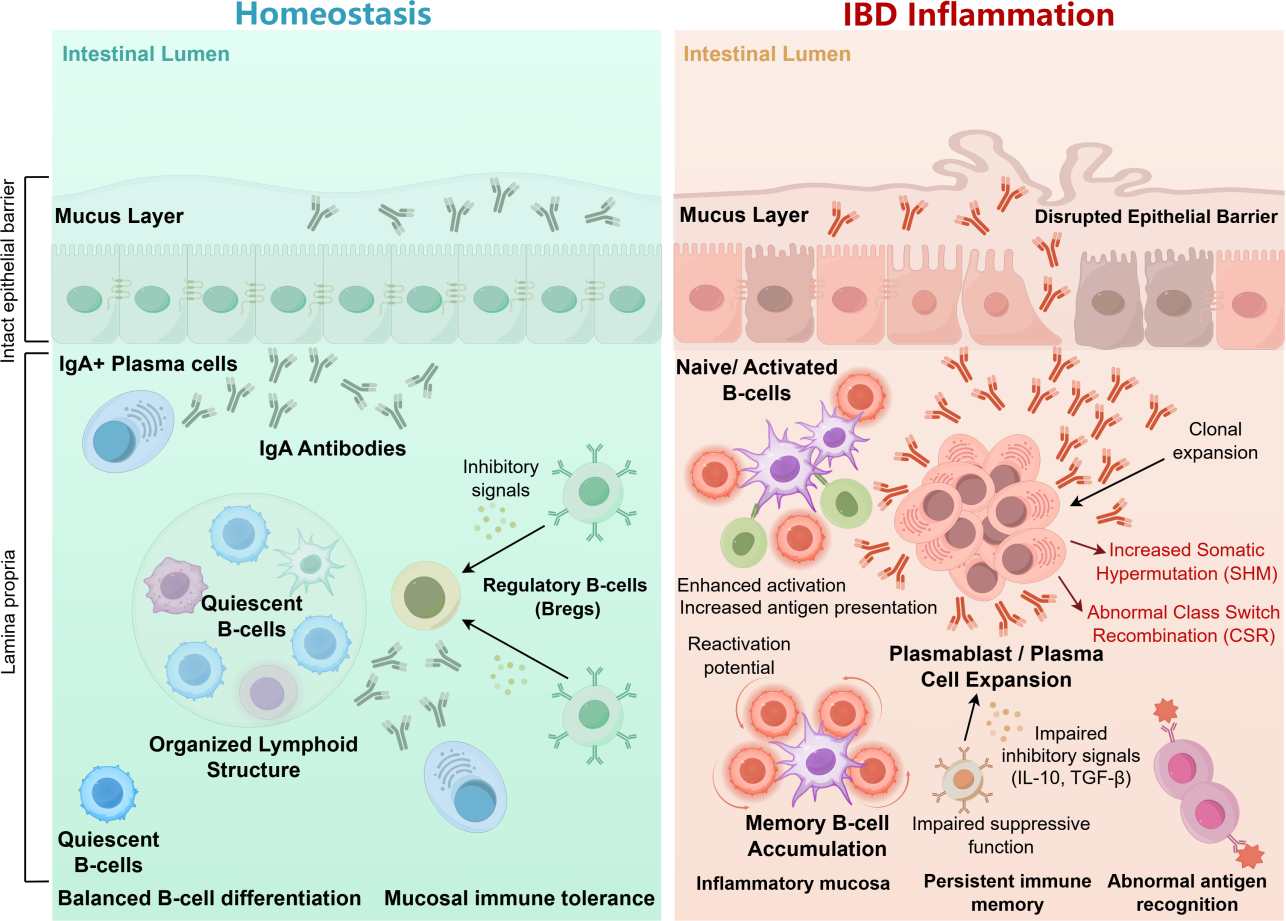
Remodeling of the B cell–humoral axis in intestinal homeostasis and IBD. The remodeling of the B cell–humoral axis in IBD evolves through a coordinated, multi-step process involving alterations in lymphoid organization, B cell differentiation, antibody output, and downstream effector responses. In the initiation phase, disruption of the epithelial barrier and increased antigen exposure promote early activation of naïve and memory B cells, while local lymphoid structures begin to reorganize. During the progression phase, sustained immune stimulation drives B cell clonal expansion, enhanced somatic hypermutation, and aberrant class switch recombination, leading to a shift in antibody composition and increased plasmablast and plasma cell differentiation. In the amplification phase, dysregulated antibody responses engage Fc receptors, complement pathways, and immune complex–mediated mechanisms, resulting in amplified inflammation and tissue injury. Importantly, these processes are interconnected and form a self-reinforcing loop linking barrier dysfunction, B cell activation, antibody remodeling, and inflammatory amplification, thereby sustaining chronic mucosal inflammation and disease progression.

The intestinal mucosa is continuously exposed to dietary antigens, commensal microbes, and their metabolites, making it one of the most immunologically active sites in the body (10). Compared with peripheral lymphoid organs, intestinal B cells exhibit a stronger degree of tissue dependency, with their distribution, activation, and differentiation tightly regulated by local antigen load, cytokine milieu, and lymphoid tissue architecture (11). In the context of IBD, the number of mucosal B cells increases and is accompanied by coordinated alterations in spatial localization, subset composition, activation status, and differentiation trajectories (12). To capture this dynamic remodeling process, we integrate current evidence from the perspectives of spatial distribution, subset reconfiguration, and functional state changes (**Figure 1**).

### 2.1 Fundamental characteristics of intestinal B cells under homeostatic conditions

Under homeostatic conditions, intestinal B cells are primarily distributed within gut-associated lymphoid tissues and the lamina propria, where IgA-producing plasma cells represent the most abundant population (11, 12). This specialized anatomical organization provides the local tissue basis for B-cell recognition of luminal antigens, interaction with T cells and dendritic cells, and subsequent differentiation into antibody-secreting cells (13). Compared with peripheral B cells, intestinal B cells are more heavily dependent on regulation by the local microenvironment, and their activation and differentiation are continuously shaped by microbial antigen load, epithelial-derived signals, stromal cell support, and cytokine networks (14, 15). In addition, a subset of intestinal B cells participates in antigen uptake and presentation, while also modulating T-cell and myeloid-cell responses through the secretion of cytokines such as IL-10, IL-6, and TGF-β (16, 17).

### 2.2 Remodeling of intestinal B cells in IBD

In IBD, intestinal B-cell remodeling represents a persistent response to profound alterations in the mucosal microenvironment. Following disruption of the epithelial barrier, microbiota-associated molecules gain increased access to the lamina propria, thereby continuously amplifying local immune stimulation (18). Concurrently, inflammatory mediators including TNF-α, IL-6, IL-21, IFN-γ, BAFF, and APRIL are markedly elevated within inflamed mucosa, leading to alterations in B-cell recruitment, survival, activation thresholds, and differentiation trajectories (19). Increased B-cell infiltration is commonly observed in patients with IBD, and in some individuals, follicle-like aggregates or ectopic lymphoid structures develop within the intestinal mucosa (20, 21), suggesting substantial changes in B-cell retention, recruitment, and local tissue organization within inflamed lesions.

Beyond alterations in spatial distribution, B cells in the inflamed intestinal mucosa of IBD patients also exhibit sustained activation and skewed differentiation. B-cell proliferation is enhanced within inflamed regions, accompanied by upregulation of activation-associated and antigen-presentation-related molecules, as well as abnormalities in clonal expansion, class-switch recombination, and somatic hypermutation (22, 23). This remodeling exerts broad amplifying effects on the intestinal immune microenvironment. Aberrantly activated B cells can influence the functions of myeloid and stromal cells through cytokine secretion, while also promoting the formation of local lymphoid-like structures that sustain inflammatory-cell accumulation (24, 25). Meanwhile, dysregulated antibody production further activates Fc receptor-, complement-, and immune complex-related pathways, thereby exacerbating inflammatory-cell recruitment and tissue injury (26). Ultimately, B-cell abnormalities interact synergistically with epithelial barrier disruption, increased microbial antigen exposure, persistent inflammatory cytokine release, and local immune structural remodeling, forming a self-reinforcing loop that perpetuates chronic intestinal inflammation in IBD.

### 2.3 Remodeling and functional reprogramming of intestinal mucosal B-cell subsets in IBD

During the course of IBD, the intestinal mucosal microenvironment undergoes persistent pathological alterations, including epithelial barrier disruption, increased exposure to microbiota-associated antigens, sustained elevation of inflammatory mediators, and remodeling of local lymphoid structures. These changes not only intensify local immune activation but also reshape the microenvironment governing B-cell recruitment, activation, differentiation, and survival. Under conditions of chronic antigenic stimulation and persistent inflammatory signaling, distinct B-cell subsets gradually undergo coordinated changes in abundance, spatial distribution, and functional state.

With the development of single-cell transcriptomic sequencing, B-cell receptor sequencing, and spatial omics technologies, the heterogeneity of intestinal mucosal B cells in IBD has been characterized with increasing resolution. Current evidence suggests that B-cell alterations in IBD are characterized not by the expansion of a single subset, but rather by the coordinated remodeling of multiple B-cell populations with distinct distributions and functional states (27, 28). Naïve/early-activated B cells, memory B cells, plasmablasts/plasma cells, regulatory B cells, and certain B-cell clones with aberrant antigen recognition all exhibit differential changes across individual patients and disease stages (29, 30). To facilitate an integrated understanding of the differences between homeostatic and inflammatory conditions, the major alterations in these B-cell subsets are summarized below (**Table 1**).

**Table 1:** Remodeling of B cell subsets in intestinal homeostasis and IBD.

B cell subset	Representative markers	Key features in homeostasis	Major alterations in IBD	References
Naïve/Early-activated B cells	CD19s-activated B cell CD86s-activa	Primarily located in gut-associated lymphoid tissues; remain in a resting or early activation state; involved in antigen recognition and initiation of differentiation	Increased abundance with enhanced activation and antigen presentation capacity, contributing to sustained local immune responses	(28, 29)
Memory B cells	CD19sy]esin IgD9 memory B cells	Maintain immunological memory following prior antigen exposure and enable rapid secondary responses	Enriched and prone to reactivation, facilitating recurrent initiation of inflammatory responses	(30, 31)
Plasmablasts/Plasma cells	CD19sablasts,onB cell su CD38^highCD138nB cell su	Predominant terminally differentiated B cell population in the lamina propria, mainly IgA-producing cells that maintain mucosal homeostasis	Markedly expanded with clonal proliferation, leading to enhanced antibody production and remodeling of local humoral immunity	(32, 33)
Regulatory B cells (Bregs)	CD19gs)oryion,nB cell IL-10s B cells	Produce immunosuppressive cytokines such as IL-10 and TGF-β, maintaining mucosal immune tolerance	Reduced in number and/or function, resulting in impaired immune regulation and insufficient control of inflammation	(34, 35)
Aberrant antigen-recognizing B cells/Expanded clones	Expanded BCR clonotypes; mutated IGHV/IGHA/IGHG	Primarily involved in physiological mucosal antigen responses under homeostatic conditions	Expanded antigen recognition spectrum, leading to abnormal responses against microbiota-derived antigens, damage-associated antigens, and self-components	(36, 37)

Among these subsets, alterations in naïve B cells and early-activated B cells reflect the persistence of local antigen-driven stimulation. Studies have identified B-cell populations in inflamed IBD mucosa characterized by upregulated activation markers and enhanced expression of antigen-presentation-related molecules (38), indicating that these cells remain in a sustained activated state. Consistent with this observation, interactions between B cells, follicular helper T cells, and dendritic cells are also enhanced (39). Changes in memory B cells are of particular importance. Animal studies have shown that memory B cells can accumulate within inflamed mucosa and associated lymphoid tissues, while retaining a strong potential for reactivation (40). Although phenotypic definitions vary across studies, the overall evidence suggests that B-cell abnormalities in IBD extend to cell populations capable of sustaining long-term immune responses.

Among the different B-cell subsets, alterations in plasmablasts and plasma cells appear to be the most consistent. Both populations are markedly increased in inflamed IBD mucosa, particularly within active lesions (41). B-cell receptor sequencing has further demonstrated that plasma-cell-associated clones are expanded in the mucosa of some patients and are accompanied by the accumulation of somatic hypermutations (42). Changes in regulatory B cells (Bregs) are more complex. Current evidence suggests that Bregs contribute to the maintenance of mucosal immune tolerance; however, both their abundance and function appear to vary in IBD (43). Some studies have reported a reduced proportion of Bregs or impaired suppressive capacity, whereas others have found that certain phenotypically defined Breg populations are not decreased and may even be relatively increased (44, 45). These discrepancies may be attributable to inconsistent definitions of Bregs, differences in marker selection, and the dependence of Breg function on the local inductive environment.

In addition, several studies have identified B-cell clones with aberrant antigen-recognition features in the intestinal mucosa of patients with IBD, including cells with reactivity toward microbiota-associated antigens, barrier-injury-associated antigens, and self-components (46, 47). The origin and functional significance of these cells remain incompletely understood; nevertheless, their presence suggests that the antigen-recognition repertoire of B cells is skewed in IBD. Remodeling of intestinal mucosal B-cell subsets in IBD is highly heterogeneous. Differences may exist between Crohn’s disease and ulcerative colitis, between the small intestine and colon, and between active and remission phases. In addition, treatment background, sampling location, and analytical approaches may all influence the interpretation of findings (48). Although single-cell sequencing, spatial omics, and B-cell receptor sequencing have improved the resolution with which B-cell heterogeneity can be characterized, several limitations remain, including inconsistent subset classification criteria, limited sample sizes, and a lack of longitudinal studies.

### 2.4 Distinct B-cell and humoral immune features in UC and CD

Although UC and CD are both classified as IBD and share common features of chronic intestinal inflammation and mucosal immune dysregulation, they exhibit distinct patterns of B-cell remodeling and humoral immune abnormalities. In UC, inflammatory lesions are primarily confined to the colonic mucosa, where the local inflammatory microenvironment is more frequently associated with enrichment of plasmablasts and plasma cells (49). Studies have demonstrated increased infiltration of CD19^+^CD27^highCD38^high plasmablasts and CD38^highCD138^+^IgG^+^ plasma cells within inflamed UC mucosa, accompanied by increased IgG-coated bacteria and enhanced IgG-associated inflammatory humoral responses (26, 50). This IgG-skewed antibody output may further amplify mucosal inflammation through Fcγ receptor activation, complement signaling, and the release of inflammatory mediators from myeloid cells (51). In addition, positivity for perinuclear anti-neutrophil cytoplasmic antibodies (pANCA) is more frequently observed in UC, suggesting a relatively characteristic humoral immune phenotype (52).

In contrast, CD is more commonly characterized by memory B-cell remodeling, persistent expansion of BCR clones, and broader anti-microbial antibody responses. Patients with CD exhibit more pronounced expansion of CD19^+^CD27^+^ memory B cells, abnormal proliferation of BCR clonal families, and increased frequencies of somatic hypermutation (53). These findings suggest that B-cell responses in CD are driven by sustained antigenic stimulation. Clinically, anti-Saccharomyces cerevisiae antibodies, anti-OmpC antibodies, and anti-CBir1 antibodies are more frequently associated with CD, potentially reflecting broad antibody responses induced by chronic exposure to microbial antigens under conditions of transmural inflammation and barrier disruption (54–56).

Taken together, the humoral immune characteristics of UC may be broadly summarized as a plasmablast/IgG-enriched mucosal humoral response, whereas CD appears to be more strongly associated with memory B-cell remodeling, persistent BCR clonal expansion, and expansion of anti-microbial antibody repertoires. Nevertheless, these differences remain influenced by disease location, inflammatory activity, therapeutic background, and methodological variability (57). Future studies integrating single-cell sequencing, spatial omics, and BCR lineage tracing will be essential to further clarify disease-specific mechanisms operating along the B-cell–humoral immunity axis in UC and CD.

## 3 Roles of humoral immunity in the intestinal immune microenvironment of IBD

**Figure 2:**
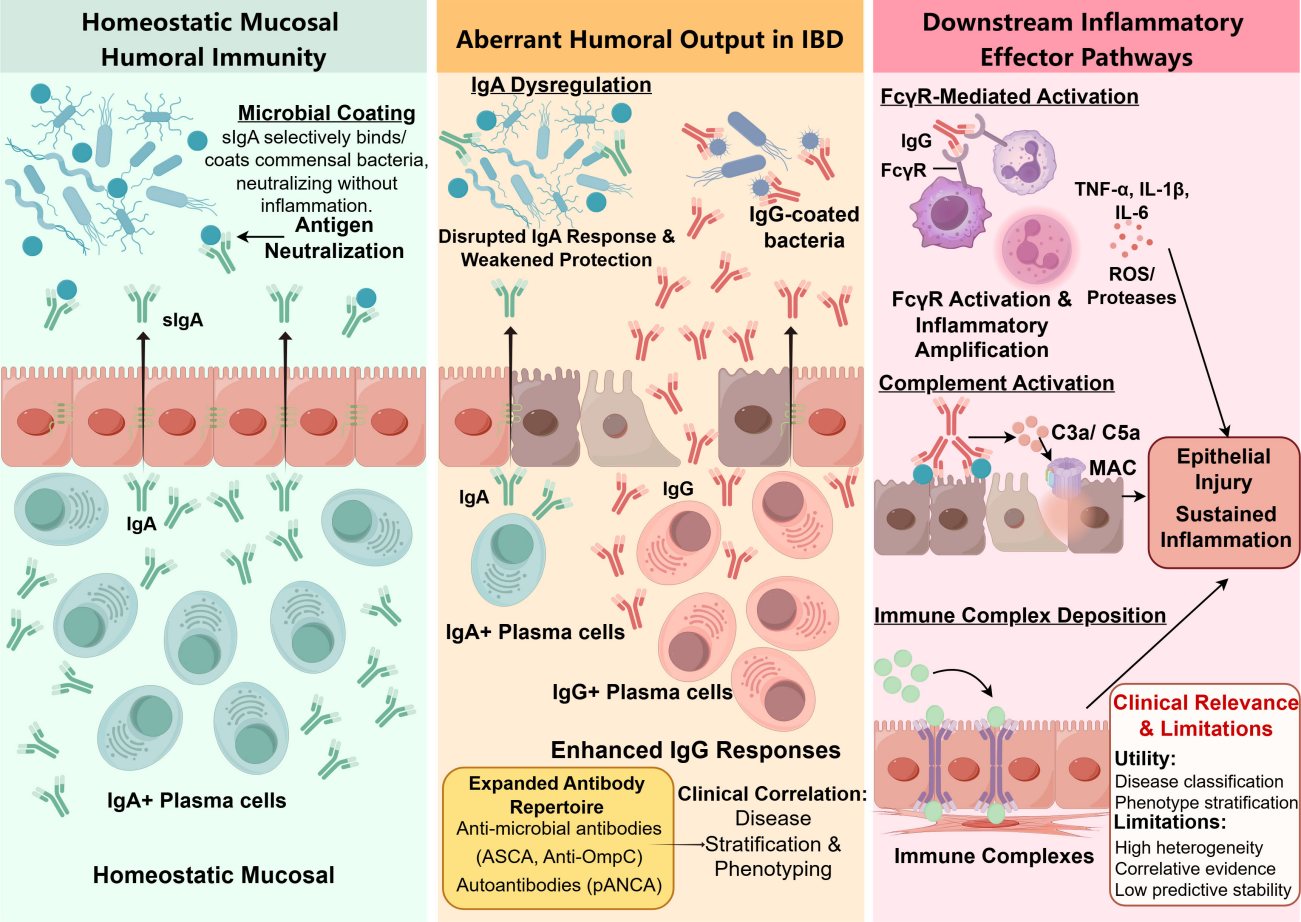
Dysregulated humoral responses drive inflammatory effector pathways in IBD. Humoral immunity in IBD undergoes a stepwise transition from mucosal protection to inflammatory amplification. Under physiological conditions, IgA antibodies mediate microbial coating and antigen neutralization, maintaining mucosal homeostasis without triggering inflammation. In IBD, this balance is disrupted, characterized by impaired IgA responses and a shift toward enhanced IgG production, leading to expanded antibody repertoires and altered microbial targeting. These changes promote Fc receptor activation, complement cascade engagement, and immune complex deposition, which collectively drive inflammatory amplification, epithelial injury, and sustained mucosal inflammation. Importantly, these interconnected events establish a self-reinforcing loop linking antibody dysregulation with chronic inflammatory damage.

Humoral immunity represents the principal effector outcome of B-cell activation and differentiation and is primarily characterized by alterations in antibody profiles together with coordinated activation of complement-, Fc receptor-, and immune complex-related responses (49, 50). In IBD, abnormalities in humoral immunity can be detected at both the mucosal and systemic levels, including altered antibody isotype distribution, increased antimicrobial and autoantibody responses, and activation of downstream effector pathways (26, 51). In addition, the formation and functional dysregulation of tertiary lymphoid structures (TLSs) may provide a localized niche for B-cell activation, clonal expansion, and skewed differentiation, thereby further promoting aberrant antibody production (52) (**Figure 2**). With the development of serological profiling, IgA-seq, B-cell receptor sequencing, and spatial omics technologies, the heterogeneity of humoral immune abnormalities in IBD has become increasingly apparent, with these alterations being influenced by disease subtype, lesion location, and inflammatory stage (53, 54).

### 3.1 Patterns of humoral immune dysregulation in IBD and their clinical relevance

Under homeostatic conditions, intestinal humoral immunity is predominantly mediated by secretory IgA (sIgA), which constitutes the major immunological output at the mucosal surface (55). Evidence suggests that sIgA limits bacterial adhesion, reduces trans-epithelial microbial translocation, and maintains spatial segregation between the microbiota and the epithelial surface, thereby preventing excessive exposure of the mucosal immune system to luminal antigens (56). In IBD, this protective humoral output becomes disrupted, manifesting as impaired IgA-associated protective functions, altered microbial coating patterns, and reduced local regulatory capacity (57, 58). Such alterations may facilitate the translocation of pro-inflammatory microbiota or microbiota-associated antigens across the mucosal barrier into the lamina propria, where they activate dendritic cells and macrophages, ultimately amplifying local inflammatory responses.

In contrast, enhancement of IgG-associated humoral responses has been more consistently observed across studies. Both serum and mucosal analyses have demonstrated elevated IgG levels, increased numbers of IgG-positive plasma cells, and expansion of IgG-coated microbiota in subsets of patients with IBD (59). Unlike IgA, which primarily contributes to mucosal tolerance and microbial containment, IgG-associated responses are more likely to promote inflammatory amplification. IgG-coated bacteria can interact with macrophages, neutrophils, and other effector cells through Fcγ receptors, thereby inducing the release of inflammatory mediators such as TNF-α, IL-1β, reactive oxygen species, and proteases, which further intensify mucosal inflammation (60–62). Concurrently, the spectrum of antimicrobial and autoantibody responses is broadened, including abnormal expression of antibodies such as ASCA, Anti-OmpC, Anti-CBir1, and pANCA (63). These antibodies not only serve as auxiliary markers for disease stratification and immune phenotyping but also suggest that antigen recognition in IBD has expanded toward a broader range of inflammation-associated humoral responses.

Beyond alterations in antibody profiles, complement-, Fc receptor-, and immune complex-associated pathways are also activated in IBD. Studies have shown that aberrant antibody responses are accompanied by enhanced Fc receptor-mediated signaling and upregulation of complement cascade components, while immune complex deposition can be detected in the intestinal tissues of some patients (64, 65). Mechanistically, binding of aberrant IgG to Fcγ receptors enhances inflammatory signal transduction in myeloid cells and promotes the release of cytokines, reactive oxygen species, and proteolytic enzymes (66). Complement activation products, particularly C3a and C5a, exert potent chemotactic and pro-inflammatory effects, thereby facilitating the recruitment of neutrophils and other inflammatory cells (67). These findings indicate that humoral immune dysregulation in IBD extends beyond antibody production itself and involves activation of multiple downstream antibody-effector pathways.

From a clinical perspective, humoral immune abnormalities are primarily relevant to disease stratification, immune phenotyping, and adjunctive disease assessment. ASCA is more commonly associated with Crohn’s disease, whereas pANCA is more frequently detected in ulcerative colitis. Antibodies such as Anti-OmpC and Anti-CBir1 may further assist in identifying specific patient subgroups (68, 69). Beyond conventional antibody panels, indices including the IgG/IgA ratio, the proportion of IgG-coated microbiota, and selected complement-related markers have also been used to evaluate the status of humoral immune output (70). Several studies have reported associations between these parameters and disease activity, the severity of mucosal inflammation, and specific disease-course phenotypes; however, these findings are substantially influenced by disease subtype, disease stage, sample source, analytical platform, and treatment background (71, 72). Accordingly, these biomarkers are more appropriately regarded as adjunctive tools for disease stratification and immune phenotyping and should be interpreted in conjunction with endoscopic, histological, and inflammatory assessments rather than being used as independent diagnostic or prognostic indicators.

Collectively, current evidence suggests that humoral immune dysregulation in IBD is primarily characterized by three major features: disruption of IgA-mediated mucosal homeostasis, expansion of IgG-associated inflammatory antibody responses and antimicrobial/autoantibody repertoires, and activation of complement-, Fc receptor-, and immune complex-related effector pathways. Evidence supporting the first two categories is relatively consistent, whereas the latter appears to be more strongly influenced by methodological differences, population heterogeneity, and disease stage, and therefore requires further mechanistic validation. From the perspective of disease subtype–specific humoral immunity, UC is more commonly characterized by a plasmablast/IgG-enriched mucosal humoral response, whereas CD exhibits more prominent features of memory B-cell remodeling, persistent BCR clonal expansion, and broadening of anti-microbial antibody repertoires, including ASCA, anti-OmpC, and anti-CBir1 antibodies.

### 3.2 Therapeutic strategies targeting humoral immune dysregulation

Humoral immune abnormalities in IBD provide several potential targets for therapeutic intervention. Current strategies mainly focus on modulating B-cell activation and differentiation, suppressing aberrant antibody production, blocking downstream antibody-effector pathways, and restoring protective mucosal humoral immunity. Among these approaches, B-cell-targeted therapies and modulation of the BAFF/APRIL signaling axis can influence B-cell survival, plasma-cell differentiation, and antibody production (73). However, because intestinal B cells also play essential roles in maintaining IgA-mediated microbial homeostasis and mucosal tolerance, broad suppression of B-cell responses may carry the risk of impairing protective immunity.

Therapeutic strategies targeting antibody-effector pathways have primarily focused on Fc receptor signaling, complement activation, and immune complex-mediated responses. In theory, inhibition of IgG-mediated Fcγ receptor activation or complement cascade signaling may reduce myeloid-cell activation, inflammatory cytokine release, and tissue injury (74). Nevertheless, clinical evidence supporting these approaches in IBD remains limited, and most current insights are derived either from mechanistic studies or from translational experience in other immune-mediated disorders.

Overall, therapeutic strategies targeting humoral immune dysregulation show considerable promise but still face several major limitations. Marked interindividual heterogeneity exists in antibody repertoires, B-cell subset composition, complement activation status, and microbiota background, making it unlikely that a single therapeutic strategy will be universally effective across all patients. Future therapeutic approaches will therefore likely depend on patient stratification, with more precise interventions selected according to IgA/IgG output patterns, antibody-profile characteristics, complement activation status, and mucosal inflammatory phenotypes.

## 4 Functional roles and mechanisms of the B cell–humoral immunity axis in IBD

In the intestinal mucosa of patients with IBD, B-cell abnormalities and humoral immune dysregulation progressively develop in parallel with alterations in local lymphoid architecture. Under inflammatory conditions, tertiary lymphoid structures (TLSs) emerge together with disruption of tissue organization, resulting in imbalance of germinal center-supporting signals. Altered CD40–CD40L, IL-21, and BAFF/APRIL signaling drives B-cell activation, clonal expansion, and differentiation, while also influencing class-switch recombination and clonal selection, thereby reshaping the composition and repertoire of antibody production (75, 76).

**Figure 3:**
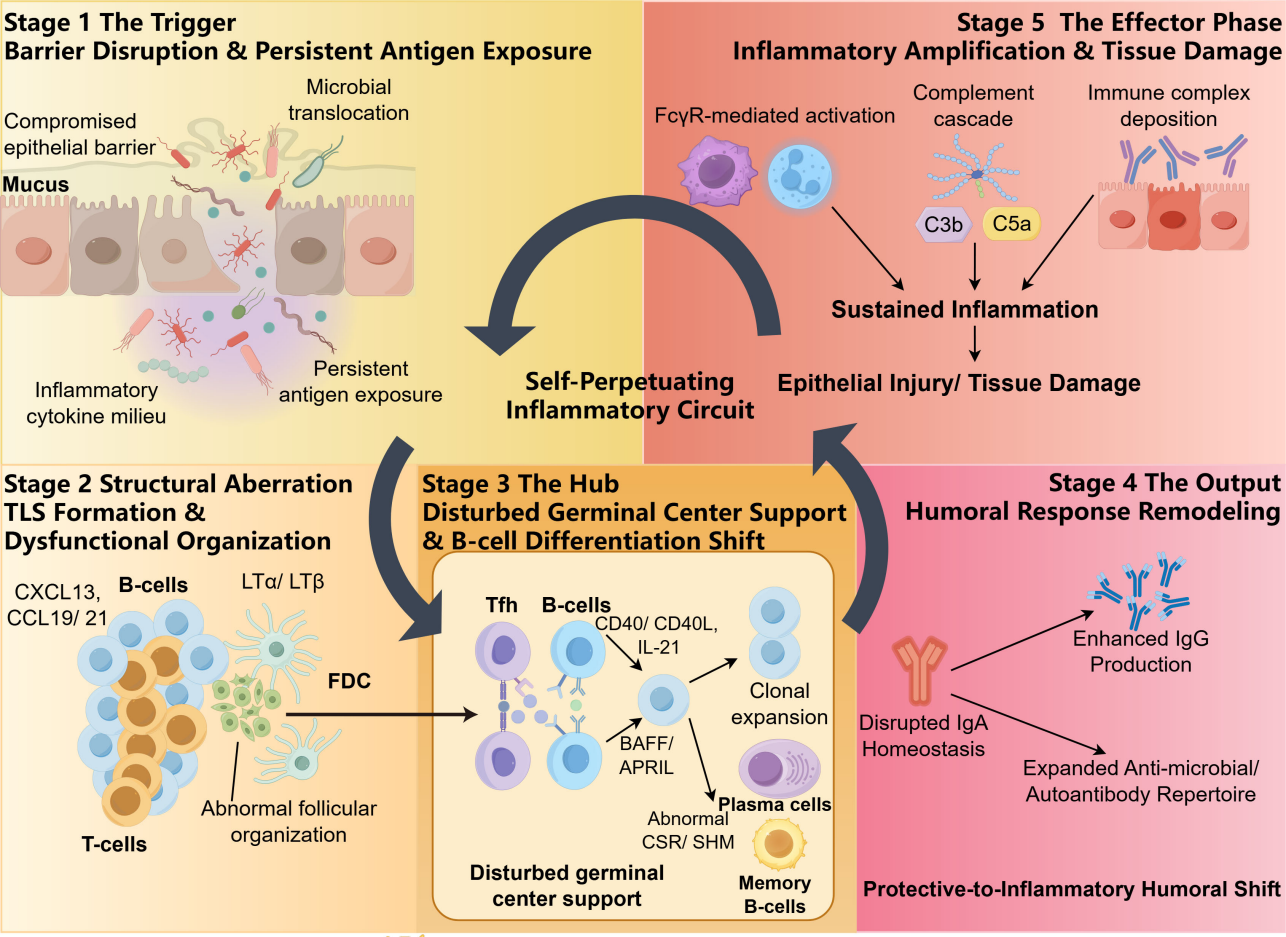
Therapeutic targeting of the B cell–humoral axis in IBD. Therapeutic modulation of the B cell–humoral axis in IBD targets multiple interconnected levels spanning lymphoid organization, B cell differentiation, antibody production, and downstream effector pathways. Upstream interventions aim to regulate ectopic lymphoid structures and reshape B cell activation and differentiation trajectories. Targeting B cells directly can suppress aberrant activation, clonal expansion, and pathogenic antibody production, while downstream strategies focus on blocking antibody-mediated effector mechanisms, including Fc receptor signaling, complement activation, and immune complex–driven inflammation. In parallel, approaches that restore protective mucosal immunity, particularly IgA-associated responses, seek to re-establish microbiota–immune homeostasis. Emerging strategies further emphasize selective modulation rather than global suppression, aiming to distinguish pathogenic from protective humoral responses. Together, these interventions outline a mechanism-based framework for patient stratification and precision immunotherapy in IBD.

On this basis, alterations in antibody output further amplify inflammatory responses. IBD is characterized by the coexistence of impaired IgA-associated mucosal protective functions and enhanced IgG-associated responses, accompanied by expansion of the antibody repertoire. Aberrant antibodies can activate immune cells through Fc receptor-mediated signaling and induce inflammatory cytokine release, amplify inflammation via complement activation, and exacerbate local tissue injury through immune complex deposition. Collectively, these processes establish an antibody-driven effector amplification loop that continuously contributes to inflammatory maintenance and epithelial damage (**Figure 3**).

### 4.1 Aberrant local lymphoid remodeling reshapes B-cell differentiation

TLSs are ectopic lymphoid-like structures frequently observed in the chronically inflamed mucosa of IBD. Their formation and maintenance depend on chemokine-mediated lymphocyte recruitment, including CXCL13, CCL19, and CCL21, as well as LTα/LTβ-associated signaling that supports local tissue organization (77). Histological and spatial omics studies have demonstrated the presence of B-cell aggregation zones, T-cell-enriched regions, and dendritic-cell networks within inflamed intestinal tissues of IBD patients. In some cases, germinal center-like structures are formed, indicating that the local microenvironment has acquired the capacity to support B-cell activation and differentiation (34, 78, 79).

TLSs in IBD are frequently accompanied by follicular structural disorganization. Severely inflamed regions often exhibit loose follicular architecture, incomplete follicular dendritic cell (FDC) networks, and impaired Tfh–B-cell interactions, thereby affecting antigen retention, clonal selection, and affinity maturation (80). Meanwhile, persistent upregulation of CD40/CD40L, IL-21, BAFF, and APRIL signaling promotes B-cell activation, survival, and plasma-cell differentiation, progressively diverting B cells away from the differentiation trajectory observed in homeostatic mucosal immunity (81).

Within this inflammatory context, activation-induced cytidine deaminase (AID)-mediated class-switch recombination (CSR) and somatic hypermutation (SHM) undergo substantial remodeling. B-cell receptor sequencing and single-cell analyses have revealed enhanced clonal expansion, mutation accumulation, and abnormal class-switching patterns in mucosal B cells from patients with IBD (82, 83). These findings suggest that aberrant local lymphoid remodeling directly reshapes B-cell selection and differentiation through alterations in chemotactic signaling, follicular support, and germinal center-associated pathways.

### 4.2 Aberrant B-cell differentiation drives inflammatory antibody responses

Under homeostatic conditions, intestinal B cells predominantly differentiate along an IgA-oriented pathway. This process is coordinately regulated by TGF-β, retinoic acid, mucosal dendritic cells, and local stromal signals, thereby promoting a low-inflammatory humoral response characterized by microbial coating and antigen neutralization (84, 85). In IBD, however, this differentiation landscape becomes profoundly altered. Following epithelial barrier disruption, microbiota-associated antigens continuously enter the mucosa, while inflammatory mediators such as IL-6, TNF-α, IL-21, and IFN-γ are markedly elevated within the local inflammatory milieu, leading to changes in B-cell activation thresholds and class-switching directionality (86, 87).

IgA-associated responses exhibit altered microbial coating patterns and impaired regulatory capacity, whereas enhancement of IgG-associated responses has been consistently observed across multiple studies, including elevated IgG levels, increased numbers of IgG-positive plasma cells, and expansion of IgG-coated microbiota (88–90). The broadening of antimicrobial and autoantibody repertoires further indicates that B-cell output shifts from maintaining mucosal homeostasis toward promoting inflammation-associated humoral responses (91). Aberrant clonal expansion and mutation accumulation further reinforce this transition. B-cell receptor sequencing studies have identified expanded B-cell clonal families and persistent somatic hypermutation in the intestinal mucosa of subsets of patients with IBD (92). Nevertheless, direct evidence establishing a causal role for these abnormal clones in disease progression remains limited.

### 4.3 Aberrant antibody-effector pathways amplify inflammation and promote tissue injury

Once aberrant antibody responses are established, multiple downstream effector pathways contribute to amplification of local inflammation. Binding of IgG and related antibodies to Fcγ receptors activates macrophages and neutrophils, thereby promoting the release of TNF-α, IL-1β, IL-6, reactive oxygen species, and proteolytic enzymes, ultimately exacerbating inflammatory-cell infiltration and tissue injury (93). Complement activation products, particularly C3a and C5a, further enhance inflammatory chemotaxis and signal amplification, while membrane attack complexes may directly contribute to epithelial damage (94). The detection of immune complex deposition within the intestinal mucosa of IBD patients further supports the existence of antibody-mediated effector injury (95).

These downstream responses, in turn, reshape the local immune microenvironment. Following epithelial barrier disruption, microbiota-associated antigens and damage-associated molecules gain access to the mucosa, thereby enhancing innate immune activation and sustaining elevated levels of IL-6, TNF-α, and BAFF signaling. This inflammatory milieu not only perpetuates myeloid-cell activation but also continuously supports B-cell activation, plasma-cell differentiation, and survival of aberrant clones (96, 97). Consequently, a continuous pathogenic cascade is established, characterized by “local structural abnormalities → skewed B-cell differentiation → amplified antibody-effector responses → persistent inflammation.”

Importantly, the strength of evidence supporting different components of this mechanistic cascade varies considerably. Increased TLS formation, B-cell aggregation, enhanced clonal expansion, elevated IgG-associated responses, and upregulation of selected complement/Fc receptor pathways have been supported by multiple population-based studies (98, 99). In contrast, the precise mechanisms through which TLS abnormalities determine B-cell fate, how aberrant antibodies directly mediate epithelial injury, and the temporal sequence of these pathogenic events during disease progression remain largely dependent on animal models and *in vitro* studies. Moreover, current evidence is predominantly derived from cross-sectional observations, whereas longitudinal studies capable of defining key transitional stages are still lacking.

## 5 Therapeutic applications and limitations of the B cell–humoral immunity axis in IBD

Interventions targeting B cells and humoral immune responses have progressively advanced from mechanistic understanding to early-stage therapeutic exploration. Unlike conventional strategies that focus on single inflammatory mediators, these approaches directly target B cell activation, antibody production, and downstream effector pathways, with the aim of modulating multiple interconnected processes, including antibody output, inflammatory amplification, and tissue injury.

Accumulating evidence indicates that B cell activation states, antibody repertoire characteristics, and associated effector pathways contribute to the persistence of inflammation in IBD and are linked to disease subtypes, relapse risk, and treatment responsiveness. These features therefore represent potential therapeutic targets (90, 91). However, most of these strategies remain in early phases of investigation, and considerable variability exists across targets in terms of strength of evidence, applicable patient populations, and safety profiles. The principal therapeutic approaches and their key characteristics are summarized in **Table 2**.

**Table 2:** Therapeutic strategies targeting the B cell–humoral axis in IBD.

Intervention level	Representative approaches	Primary targeted processes	Potential clinical implications	Research stage	References
TLS modulation	Chemokine-targeting strategies (e.g., CXCL13-related pathways)	Regulation of ectopic lymphoid structure formation and germinal center responses	Modulation of B cell differentiation trajectories and antibody class distribution	Preclinical research	(100–102)
B cell targeting	Anti-CD20 therapy; BAFF/APRIL-targeting strategies	Inhibition of aberrant B cell activation, survival, and clonal expansion	Reduction of pathogenic B cell burden and antibody production	Early clinical investigation	(103, 104)
Antibody-directed interventions	IgG neutralization; Fc receptor blockade	Suppression of antibody-mediated myeloid cell activation	Attenuation of inflammatory cell infiltration and tissue damage	Early-stage research	(105–107)
Complement modulation	C3/C5 pathway inhibition	Inhibition of complement-mediated chemotaxis and membrane attack complex formation	Reduction of inflammatory amplification and epithelial injury	Limited evidence in IBD	(108–110)
Mucosal immune modulation	IgA induction strategies; mucosa-targeted immunoregulation	Enhancement of protective mucosal antibody responses	Restoration of microbiota–immune homeostasis and barrier function	Exploratory stage	(111–113)
Engineered strategies	Engineered B cells; engineered antibodies	Precise modulation of B cell differentiation or antibody specificity	Improved targeting precision of immunomodulation	Preclinical and emerging research	(114, 115)

### 5.1 Therapeutic applications of the B cell–humoral immunity axis in IBD

The multilayered involvement of the B cell–humoral immunity axis in IBD provides a well-defined framework for therapeutic intervention. In terms of mechanistic level, current strategies can be broadly divided into three categories: approaches that regulate the microenvironment governing B-cell activation and differentiation, thereby influencing humoral immune development upstream; approaches that directly target B cells to limit aberrant clonal expansion and antibody production; and approaches that act on antibodies or their downstream effector pathways to attenuate Fc receptor-, complement-, and immune complex-mediated inflammatory responses (116, 117).

At the upstream level, TLSs and their associated chemotactic signals represent potential therapeutic targets. The formation of TLSs, maintenance of follicular architecture, and germinal center responses participate in B-cell selection, class-switch recombination, and plasma-cell differentiation. Therefore, modulation of chemokines such as CXCL13 or of the local inflammatory milieu may influence the subsequent structure of antibody output (118). The distinctive feature of this strategy is that it seeks to reshape B-cell fate at the level of the local microenvironment. However, current evidence is largely derived from animal models and mechanistic studies, with limited human data demonstrating direct improvement in clinical outcomes in IBD. Accordingly, TLS modulation remains at the stage of mechanistic validation.

Direct B-cell-targeted strategies are more practically actionable, with anti-CD20 therapy and modulation of the BAFF/APRIL pathway representing the main areas of investigation. These interventions may reduce B-cell activation, survival, and clonal expansion, thereby lowering the burden of aberrant antibody production (119). However, their efficacy in IBD has been inconsistent. This is because intestinal B cells do not exert purely pro-inflammatory effects; certain subsets and IgA-associated plasma cells contribute to microbiota homeostasis, barrier function, and mucosal tolerance (120, 121). Broad depletion of B cells may therefore simultaneously impair protective mucosal immunity, making the balance between therapeutic benefit and risk more complex.

At the level of antibodies and their downstream effector pathways, interventions mainly focus on IgG-associated responses, Fc receptor signaling, and the complement pathway (122). IgG can mediate myeloid-cell activation, while complement activation further amplifies inflammatory responses; thus, blockade of these pathways has a clear mechanistic rationale (123, 124). Complement modulation has already been applied in other immune-mediated diseases (125) and may hold translational potential in IBD. Nevertheless, relevant studies remain limited, and stable population-level evidence supporting efficacy is still lacking. Moreover, interindividual differences in antibody repertoires and complement activation status may restrict the broad applicability of these strategies.

Another therapeutic approach does not aim to suppress aberrant humoral immunity directly but instead seeks to restore protective mucosal immunity. Promoting IgA production, improving microbial coating, and enhancing mucosal immune homeostasis may help re-establish a low-inflammatory humoral immune environment (126, 127). This strategy more closely resembles physiological immune regulation, but its efficacy may be influenced by local antigen load, cytokine context, and microbiota composition (128). Its clinical stability and long-term effects therefore require further evaluation.

In addition, some therapies that do not directly target B cells or antibodies may still be accompanied by changes in humoral immunity. For example, anti-TNF-α therapy may reduce aberrant antibody responses or remodel antibody repertoires while suppressing inflammation (129). This observation suggests that humoral immunity may not only participate in disease activity but also contribute to therapeutic response (130). However, this association is currently based mainly on concomitant changes, and its causal relevance and stability require further validation.

### 5.2 Challenges and future opportunities for clinical translation

The clinical translation of B cell–humoral immunity-targeted strategies in IBD is first limited by the functional heterogeneity of B cells (131). Different subsets play distinct roles during inflammation and may even exert functionally opposing effects. However, tools capable of reliably distinguishing and selectively modulating these subsets *in vivo* are still lacking (132). This issue is particularly important in IBD. Although broad suppression of B cells or antibody responses may reduce aberrant immune activation, it may also weaken IgA-associated protective mucosal immunity (133), increasing the risk of barrier dysfunction and infection and thereby limiting the therapeutic window of such strategies.

Interindividual differences in immune phenotypes further complicate clinical translation. Antibody repertoires, complement activation status, immune complex formation, and B-cell subset distribution are influenced by multiple factors, including microbiota composition, disease stage, lesion location, and previous treatments (134, 135). As a result, the immunological basis for the same intervention may differ substantially across patients. The current lack of stable and generalizable patient-stratification tools has become a major barrier to clinical application in this field.

In addition, most available evidence is derived from cross-sectional studies, small exploratory cohorts, and model-based validation. Although some targets have a clear mechanistic rationale, their long-term efficacy, safety, and appropriate target populations remain uncertain. For chronic relapsing diseases such as IBD, therapeutic strategies must simultaneously account for inflammatory control, infection risk, and preservation of mucosal immune homeostasis (136). In this context, advancing clinical application based solely on mechanistic plausibility remains clearly insufficient.

### 5.3 Emerging research directions for the B cell–humoral immunity axis in IBD

Research on the B cell–humoral immunity axis is reshaping the conceptual framework of the mucosal immune microenvironment in IBD and is gradually extending toward the development of diagnostic and therapeutic strategies (137, 138). The introduction of multi-omics technologies has enabled higher-resolution characterization of B-cell differentiation states, antibody repertoire architecture, and their relationships with the microbiota and local inflammatory networks (139, 140). Current evidence suggests that B cells and antibody responses are involved in multiple processes, including microbiota–immune interactions, maintenance of the mucosal barrier, and persistence of inflammation, and are closely associated with heterogeneity in therapeutic responses. These findings have shifted the B cell–humoral immunity axis from an auxiliary component to a core element that can be systematically evaluated and therapeutically targeted.

Future studies are likely to focus more closely on the dynamic behavior of B cells within the local mucosal environment, including the spatial localization, differentiation trajectories, and clonal evolution of B-cell subsets, as well as their interactions with epithelial cells, myeloid cells, and the microbiota. Compared with earlier studies that mainly focused on B-cell numbers or antibody levels, current research is increasingly shifting toward the functional significance of specific subsets, antibody isotypes, and local lymphoid structures across different disease stages. This shift will help clarify the hierarchical roles of B cell–humoral immunity in IBD and provide a basis for identifying key regulatory nodes.

Antibody-related research is also moving toward a systems-level framework. Current areas of interest include the distribution patterns of IgA and IgG, the functional differences between antimicrobial antibodies and autoantibodies, and the relevance of these features to disease classification, relapse-risk assessment, and therapeutic response. At the same time, direct evidence remains limited regarding the precise mechanisms through which antibodies contribute to mucosal injury, inflammatory amplification, and local immune regulation. Functional studies are therefore urgently needed. Systematic characterization of antibody features will help establish a more interpretable framework for immune phenotyping.

In addition, interactions between B cell–humoral immunity and other immune or tissue components will become an important focus of future research. Through antigen presentation, cytokine secretion, and antibody production, B cells regulate the functions of Tfh cells, Treg cells, macrophages, and epithelial cells, while antibody- and complement-mediated responses further participate in inflammatory-cell recruitment and tissue injury (141, 142). Systematic analysis of these interactions will help reveal how the immune network is globally reorganized in IBD and will provide a structural basis for multi-target intervention.

### 5.4 Exploration of novel immune targets and therapeutic strategies

Interventional targets based on the B cell–humoral immunity axis are gradually being defined. With deeper understanding of B-cell differentiation, germinal center reactions, and plasma-cell maintenance, the BAFF/APRIL axis, BCMA, and Tfh-related signals have increasingly become key areas of investigation. These molecules are directly involved in B-cell survival, differentiation, and antibody production, providing a clear mechanistic basis for therapeutic intervention.

The key to intervention is not broad suppression of B-cell responses, but rather the distinction between pathogenic and protective subsets and the implementation of selective modulation. Existing studies indicate that different B-cell populations exert markedly divergent roles in IBD (143). Some IgG-associated plasma cells, autoreactive B cells, and persistently expanded abnormal clones are more likely to participate in inflammatory maintenance and tissue injury (144, 145), whereas IgA^+^ plasma cells and certain regulatory B cells contribute to microbiota homeostasis and mucosal tolerance (146). Therefore, strategies with greater translational potential should aim to limit pathogenic humoral immunity while preserving protective mucosal immunity.

Interventions targeting antibodies and their downstream effector pathways also deserve attention. Modulation of aberrant IgG, autoantibodies, Fc receptor signaling, and complement activation may help reduce inflammatory-cell recruitment and immune complex-associated tissue injury (147, 148). Meanwhile, restoration of IgA-dominated mucosal humoral immunity may enhance microbial coating and maintain local tolerance, thereby approximating a more physiological mode of immune regulation (149, 150). However, the stability and long-term efficacy of these strategies across different inflammatory contexts remain to be validated.

Engineered B cells and engineered antibodies represent more advanced directions for therapeutic intervention. Gene-editing and cellular-engineering technologies may enable targeted regulation of B-cell antigen recognition, antibody production, and cytokine secretion, thereby allowing more precise immune modulation. Although these strategies have been explored in other diseases, their application in IBD remains at an early stage. Overall, interventions related to B cell–humoral immunity are moving toward patient stratification and subset-specific targeting.

### 5.5 Technological advances driving B cell–humoral immunity research

Technological advances continue to drive research on B cell–humoral immunity from descriptive analysis toward mechanistic dissection. Early studies primarily relied on histology, flow cytometry, and serological assays (151). In recent years, the application of high-dimensional technologies has advanced the field toward higher cellular and spatial resolution. Transcriptomic sequencing and B-cell receptor sequencing have provided key tools for dissecting B-cell clonal expansion and antibody diversity (152). Through lineage analysis, it is possible to identify the expansion of specific clones in inflamed mucosa, their mutation accumulation, and their dynamic changes during disease progression.

Single-cell technologies have further improved resolution. Single-cell RNA sequencing combined with BCR sequencing can distinguish different B-cell subsets and characterize their activation states, antibody isotype biases, and relationships with the local cytokine environment (153). Such information helps explain interpatient differences in humoral immune response patterns.

Spatial transcriptomics and multiplex imaging technologies provide spatial information on local immune responses. IBD lesions exhibit marked regional heterogeneity, with uneven distributions of epithelial injury, immune infiltration, and lymphoid structures across different areas (154). Spatial technologies can reveal the spatial relationships among B cells, plasma cells, and inflammatory regions, thereby defining key sites of antibody production and inflammatory amplification. At the same time, high-throughput antibody profiling, proteomics, and mass spectrometry have made systematic analysis of antibody repertoires increasingly feasible. When combined with artificial intelligence and machine-learning approaches, single-cell, spatial omics, microbiome, and clinical data can be integrated for patient stratification and prediction of therapeutic response. This integrative analytical framework is expected to improve the precision of immune phenotyping in IBD.

## 6 Conclusion

The B cell–humoral immunity axis represents a fundamental component of mucosal immune dysregulation in IBD. Incorporating this axis into the broader framework of local immune regulation enables a more comprehensive understanding of IBD immunopathogenesis. Current evidence supports that this axis does not operate in isolation, but rather unfolds as a continuous, interconnected process involving alterations in local lymphoid architecture, shifts in B cell differentiation, remodeling of the antibody repertoire, and activation of Fc receptor–, complement-, and immune complex–mediated effector pathways.

Within this framework, TLS abnormalities reshape the microenvironment governing B cell activation and differentiation, differentiation bias drives changes in antibody output, and aberrant antibodies together with their downstream effector mechanisms contribute to inflammatory amplification and tissue injury. A key priority for future research is to delineate the hierarchical roles of this mechanistic sequence across different IBD subtypes and disease stages, and to distinguish pathogenic from protective humoral immune responses.

Building on this foundation, the integration of patient stratification strategies and longitudinal study designs will be essential to transition the B cell–humoral immunity axis from a descriptive mechanistic concept to a clinically actionable immunophenotyping tool and a basis for selective therapeutic intervention.

## References

[1] Haque PS Kapur N Barrett TA Theiss AL . Mitochondrial function and gastrointestinal diseases. Nat Rev Gastroenterol Hepatol. (2024) 21:537–55. doi: 10.1038/s41575-024-00931-2 38740978 PMC12036329

[2] Kucharzik T Taylor S Allocca M Burisch J Ellul P Iacucci M . ECCO-ESGAR-ESP-IBUS guideline on diagnostics and monitoring of patients with inflammatory bowel disease: Part 1. J Crohns Colitis. (2025) 19:jjaf106. doi: 10.1093/ecco-jcc/jjaf106 40741688

[3] Zheng L Duan SL Wang K . Research progress concerning the involvement of the intestinal microbiota in the occurrence and development of inflammatory bowel disease. World J Gastroenterol. (2025) 31:113170. doi: 10.3748/wjg.v31.i42.113170 41278154 PMC12635724

[4] Eden N Gaunt E Ong EMS Sharif K Selinger C . The role of novel small molecule drugs in the management of inflammatory bowel disease. Br J Hosp Med (Lond). (2025) 86:1–14. doi: 10.12968/hmed.2024.0798 40554447

[5] Huang X Huang Y . Recent progress of novel strategies of small molecule drugs to treat inflammatory bowel diseases. Bioorg Chem. (2026) 171:109535. doi: 10.1016/j.bioorg.2026.109535 41619437

[6] Fan JN Ho H Chiang BL . Characterization of novel CD8+ regulatory T cells and their modulatory effects in murine model of inflammatory bowel disease. Cell Mol Life Sci. (2024) 81:327. doi: 10.1007/s00018-024-05378-x 39085655 PMC11335251

[7] Castillo FA Kern BC Villablanca EJ . B cells in inflammatory bowel disease. Immunol Lett. (2026) 277:107071. doi: 10.1016/j.imlet.2025.107071 40845931

[8] Chen D Xu S Li S Wang Q Li H He D . The multi-organ landscape of B cells highlights dysregulated memory B cell responses in Crohn's disease. Natl Sci Rev. (2025) 12:nwaf009. doi: 10.1093/nsr/nwaf009 40160682 PMC11951101

[9] Fung ICN Koelink PJ Mulders LGM Admiraal I Verseijden C Verhoeff J . Mucosal B cell expansion and maturation contribute to colitis pathogenesis. Inflammation Bowel Dis. (2026) 32:290–302. doi: 10.1093/ibd/izaf275 41267169 PMC12857420

[10] Takeuchi T Nakanishi Y Ohno H . Microbial metabolites and gut immunology. Annu Rev Immunol. (2024) 42:153–78. doi: 10.1146/annurev-immunol-090222-102035 38941602

[11] Siniscalco ER Meng H Gabernet G Pacheco GA Saghaei S Ramirez SI . Sequential class switching generates antigen-specific gut IgA from IgG1 B cells. Immunity. (2025) 58:3075–3093.e6. doi: 10.1016/j.immuni.2025.10.022 41253159 PMC12834128

[12] Yao X Ma K Zhu Y Cao S . Innate lymphoid cells in inflammatory bowel disease. Cells. (2025) 14:825. doi: 10.3390/cells14110825 40498001 PMC12155180

[13] Pernes JI Alsayah A Tucci F Bashford-Rogers RJM . Unravelling B cell heterogeneity: insights into flow cytometry-gated B cells from single-cell multi-omics data. Front Immunol. (2024) 15:1380386. doi: 10.3389/fimmu.2024.1380386 38707902 PMC11067501

[14] Bukhari AM Meng W Rosenfeld AM Prak ETL Knight KL . IgM+IgD- B cells in human gut-associated lymphoid tissue have memory features and give rise to IgM+ and IgA+ antibody-secreting cells. Sci Rep. (2025) 15:26613. doi: 10.1038/s41598-025-11209-1 40695977 PMC12284196

[15] Bemark M Pitcher MJ Dionisi C Spencer J . Gut-associated lymphoid tissue: a microbiota-driven hub of B cell immunity. Trends Immunol. (2024) 45:211–23. doi: 10.1016/j.it.2024.01.006 38402045 PMC11227984

[16] Zogorean R Wirtz S . The yin and yang of B cells in a constant state of battle: intestinal inflammation and inflammatory bowel disease. Front Immunol. (2023) 14:1260266. doi: 10.3389/fimmu.2023.1260266 37849749 PMC10577428

[17] Wiarda JE Shircliff AL Becker SR Stasko JB Sivasankaran SK Ackermann MR . Conserved B cell signaling, activation, and differentiation in porcine jejunal and ileal Peyer's patches despite distinct immune landscapes. Mucosal Immunol. (2024) 17:1222–41. doi: 10.1016/j.mucimm.2024.08.005 39147277

[18] Kampe L Melcher C Westphal K Brand K Föger N Lee KH . Acquisition of innate B cell properties and generation of autoreactive IgA antibodies by follicular B cells during homeostatic proliferation. Front Immunol. (2025) 16:1506628. doi: 10.3389/fimmu.2025.1506628 39911392 PMC11794109

[19] Zheremyan EA Ustiugova AS Uvarova AN Karamushka NM Stasevich EM Gogoleva VS . Differentially activated B cells develop regulatory phenotype and show varying immunosuppressive features: a comparative study. Front Immunol. (2023) 14:1178445. doi: 10.3389/fimmu.2023.1178445 37731503 PMC10509016

[20] Li H Zhang Y Du S Shen J Liu X Jing J . Remodeling the intestinal immune microenvironment": immune regulation and tissue regeneration by mesenchymal stem/stromal cells in the repair microenvironment of inflammatory bowel disease. Front Immunol. (2025) 16:1543702. doi: 10.3389/fimmu.2025.1543702 40433382 PMC12106535

[21] Kotagiri P Rae WM Bergamaschi L Pombal D Lee JY Noor NM . Disease-specific B cell clones are shared between patients with Crohn's disease. Nat Commun. (2025) 16:3689. doi: 10.1038/s41467-025-58977-y 40246842 PMC12006383

[22] Abbaszadeh M Naseri B Taghizadeh-Teymorloei M Mardi A Javan MR Masoumi J . Overview of dendritic cells subsets and their involvement in immune-related pathological disease. Bioimpacts. (2025) 15:30671. doi: 10.34172/bi.30671 40256217 PMC12008504

[23] Chien CH Yeh TY Chiang BL . Non-antigen-specific B cells induced regulatory CD4+ T cells through decreasing T cell activation. Immunology. (2025) 175:434–43. doi: 10.1111/imm.13940 40320632

[24] Brown M Dodd A Shi F Greenwood E Nagpal S Kolachala VL . Concordant B and T cell heterogeneity inferred from the multiomic landscape of peripheral blood mononuclear cells in a Crohn's disease cohort. J Crohns Colitis. 18:1939–56. doi: 10.1093/ecco-jcc/jjae055 38613150 PMC11637485

[25] Kilian C Ulrich H Zouboulis VA Sprezyna P Schreiber J Landsberger T . Longitudinal single-cell data informs deterministic modelling of inflammatory bowel disease. NPJ Syst Biol Appl. (2024) 10:69. doi: 10.1038/s41540-024-00395-9 38914538 PMC11196733

[26] Uzzan M Martin JC Mesin L Livanos AE Castro-Dopico T Huang R . Ulcerative colitis is characterized by a plasmablast-skewed humoral response associated with disease activity. Nat Med. (2022) 28:766–79. doi: 10.1038/s41591-022-01680-y 35190725 PMC9107072

[27] Veh J Ludwig C Schrezenmeier H Jahrsdörfer B . Regulatory B cells-immunopathological and prognostic potential in humans. Cells. (2024) 13:357. doi: 10.3390/cells13040357 38391970 PMC10886933

[28] Sharma R Smolkin RM Chowdhury P Fernandez KC Kim Y Cols M . Distinct metabolic requirements regulate B cell activation and germinal center responses. Nat Immunol. (2023) 24:1358–69. doi: 10.1038/s41590-023-01540-y 37365386 PMC11262065

[29] He X Zhao J Adilijiang A Hong P Chen P Lin X . Dhx33 promotes B-cell growth and proliferation by controlling activation-induced rRNA upregulation. Cell Mol Immunol. (2023) 20:277–91. doi: 10.1038/s41423-022-00972-0 36631557 PMC9970960

[30] Kirchenbaum GA Pawelec G Lehmann PV . The importance of monitoring antigen-specific memory B cells, and how ImmunoSpot assays are suitable for this task. Cells. (2025) 14:223. doi: 10.3390/cells14030223 39937014 PMC11816810

[31] Inoue T Kurosaki T . Memory B cells. Nat Rev Immunol. (2024) 24:5–17. doi: 10.1038/s41577-023-00897-3 37400644

[32] Habenicht KM Wanzek J Bootz A Schäfer S Vollmer L Hiergeist A . Expansions of circulating plasmablasts producing commensal-reactive IgA antibodies are predictors for chronic GVHD. Blood. (2025) 145:3178–88. doi: 10.1182/blood.2024027301 40324063

[33] Pracht K Wittner J Kagerer F Jäck HM Schuh W . The intestine: a highly dynamic microenvironment for IgA plasma cells. Front Immunol. (2023) 14:1114348. doi: 10.3389/fimmu.2023.1114348 36875083 PMC9977823

[34] Gauthier J Maugendre M Léonard S Desvois Y Pineau M Pinon R . B cell-intrinsic IL-2 signaling regulates inflammation by promoting IL-10 expression in CD25+ age-associated B cells. Immunity. (2026) 59:354–371.e9. doi: 10.1016/j.immuni.2026.01.015 41619728

[35] Memida T Abdolahinia ED Cao G Ruiz S Huang S Shindo S . B10 cells promote pro-resolving macrophage function through direct cell-cell contact and IL-10 secretion in Raw 264.7 cells. Int Immunol. (2025) 37:457–74. doi: 10.1093/intimm/dxaf012 40056161 PMC12641531

[36] Steiner TM Kato Y Tan PS Tullett KM Panetta F Davey GM . MHC II-restricted presentation of soluble antigen by naïve B cells is impaired upon engagement with membrane-associated antigen: a potential mechanism to mitigate autoreactivity. J Immunol. (2025) 214:2189–201. doi: 10.1093/jimmun/vkaf129 40581630

[37] Prior JT Limbert VM Horowitz RM D'Souza SJ Bachnak L Godwin MS . Establishment of isotype-switched, antigen-specific B cells in multiple mucosal tissues using non-mucosal immunization. NPJ Vaccines. (2023) 8:80. doi: 10.1038/s41541-023-00677-z 37258506 PMC10231862

[38] Scheid JF Eraslan B Hudak A Brown EM Sergio D Delorey TM . Remodeling of colon plasma cell repertoire within ulcerative colitis patients. J Exp Med. (2023) 220:e20220538. doi: 10.1084/jem.20220538 36752797 PMC9949229

[39] Sollid LM Iversen R . Tango of B cells with T cells in the making of secretory antibodies to gut bacteria. Nat Rev Gastroenterol Hepatol. (2023) 20:120–8. doi: 10.1038/s41575-022-00674-y 36056203

[40] Reza Lahimchi M Eslami M Yousefi B . Interleukin-35 and interleukin-37 anti-inflammatory effect on inflammatory bowel disease: application of non-coding RNAs in IBD therapy. Int Immunopharmacol. (2023) 117:109932. doi: 10.1016/j.intimp.2023.109932 37012889

[41] Spencer J Bemark M . Human intestinal B cells in inflammatory diseases. Nat Rev Gastroenterol Hepatol. (2023) 20:254–65. doi: 10.1038/s41575-023-00755-6 36849542

[42] Liu J Lai X Bao Y Xie W Li Z Chen J . Intraperitoneally delivered mesenchymal stem cells alleviate experimental colitis through THBS1-mediated induction of IL-10-competent regulatory B cells. Front Immunol. (2022) 13:853894. doi: 10.3389/fimmu.2022.853894 35371051 PMC8971528

[43] Mahdy AKH Taheri Z Høivik ML Franke A ElAbd H . High throughput profiling of the B cell repertoire identifies systematic changes in the repertoire of individuals with Crohn's disease. Front Immunol. (2026) 17:1725813. doi: 10.3389/fimmu.2026.1725813 41727424 PMC12920447

[44] Shen X Mo S Zeng X Wang Y Lin L Weng M . Identification of antigen-presentation related B cells as a key player in Crohn's disease using single-cell dissecting, hdWGCNA, and deep learning. Clin Exp Med. (2023) 23:5255–67. doi: 10.1007/s10238-023-01145-7 37550553

[45] Gomez-Bris R Saez A Herrero-Fernandez B Rius C Sanchez-Martinez H Gonzalez-Granado JM . CD4 T-cell subsets and the pathophysiology of inflammatory bowel disease. Int J Mol Sci. (2023) 24:2696. doi: 10.3390/ijms24032696 36769019 PMC9916759

[46] Bai Y Ding J He L Zhu Z Pan J Qi C . β-Glucan induced plasma B cells differentiation to enhance antitumor immune responses by Dectin-1. BMC Immunol. (2025) 26:2. doi: 10.1186/s12865-025-00681-z 39794756 PMC11724571

[47] Liu Z Dai H Cui X Liu Y Dong Z . Importance of B cells (review). Int J Mol Med. (2026) 57:2. doi: 10.3892/ijmm.2025.5673 41133471 PMC12576395

[48] Yue N Hu P Tian C Kong C Zhao H Zhang Y . Dissecting innate and adaptive immunity in inflammatory bowel disease: Immune compartmentalization, microbiota crosstalk, and emerging therapies. J Inflammation Res. (2024) 17:9987–10014. doi: 10.2147/JIR.S492079 39634289 PMC11615095

[49] Uo M Hisamatsu T Miyoshi J Kaito D Yoneno K Kitazume MT . Mucosal CXCR4+ IgG plasma cells contribute to the pathogenesis of human ulcerative colitis through FcγR-mediated CD14 macrophage activation. Gut. (2013) 62:1734–44. doi: 10.1136/gutjnl-2012-303063 23013725

[50] Wang X Jiang Y Zhu Y Zhang M Li M Wang H . Circulating memory B cells and plasmablasts are associated with the levels of serum immunoglobulin in patients with ulcerative colitis. J Cell Mol Med. (2016) 20:804–14. doi: 10.1111/jcmm.12728 26800315 PMC4831367

[51] Hematianlarki M Nimmerjahn F . Immunomodulatory and anti-inflammatory properties of immunoglobulin G antibodies. Immunol Rev. (2024) 328:372–86. doi: 10.1111/imr.13404 39340138 PMC11659946

[52] Kim JM Choi YM Jung SA Yang HR . Diagnostic utility, disease activity, and disease phenotype correlation of serum ASCA, pANCA, and PR3-ANCA in pediatric inflammatory bowel disease. J Pediatr (Rio J). (2024) 100:204–11. doi: 10.1016/j.jped.2023.10.005 38012956 PMC10943302

[53] Wang Y Zhao R Liang Q Ni S Yang M Qiu L . Organ-based characterization of B cells in patients with systemic lupus erythematosus. Front Immunol. (2025) 16:1509033. doi: 10.3389/fimmu.2025.1509033 39917309 PMC11798990

[54] Lisai-Goldstein Y Focht G Orlanski-Meyer E Yogev D Lev-Tzion R Ledder O . Serological markers as predictors of anti-TNF response in children with Crohn's disease. Dig Dis Sci. (2025) 70:333–9. doi: 10.1007/s10620-024-08732-y 39604667

[55] Ibrahim MK Cohen R Chhibba T Kumar M Lau H McGovern D . An international multicenter study of native and immigrant South Asian Crohn's disease. Clin Gastroenterol Hepatol. (2026) 24:1121–1129.e6. doi: 10.1016/j.cgh.2025.06.022 40633890

[56] Shome M Song L Williams S Chung Y Murugan V Park JG . Serological profiling of Crohn's disease and ulcerative colitis patients reveals anti-microbial antibody signatures. World J Gastroenterol. (2022) 28:4089–101. doi: 10.3748/wjg.v28.i30.4089 36157118 PMC9403437

[57] Statie RC Florescu DN Gheonea DI Ungureanu BS Iordache S Rogoveanu I . The use of endoscopic ultrasonography in inflammatory bowel disease: A review of the literature. Diagnostics (Basel). (2023) 13:568. doi: 10.3390/diagnostics13030568 36766671 PMC9914551

[58] Puechberty S Sterlin D . The antibody-microbiota interface in autoimmune diseases. Muscle Nerve. (2026) 1–17. doi: 10.1002/mus.70131 41517962

[59] Bamias G Kitsou K Rivera-Nieves J . The underappreciated role of secretory IgA in IBD. Inflammation Bowel Dis. (2023) 29:1327–41. doi: 10.1093/ibd/izad024 36943800 PMC10393212

[60] Preto AJ Chanana S Ence D Healy MD Domingo-Fernández D West KA . Multi-omics data integration identifies novel biomarkers and patient subgroups in inflammatory bowel disease. J Crohns Colitis. (2025) 19:jjae197. doi: 10.1093/ecco-jcc/jjae197 39756419 PMC11792892

[61] Liu L Davidorf B Dong P Peng A Song Q He Z . Decoding the mosaic of inflammatory bowel disease: Illuminating insights with single-cell RNA technology. Comput Struct Biotechnol J. (2024) 23:2911–23. doi: 10.1016/j.csbj.2024.07.011 39421242 PMC11485491

[62] Ando M Kito I Rachi T Matsuda T Oshima K . Formation of the intestinal microbiota during mouse weaning promotes maturation of the IgA repertoire after growth. Biosci Microbiota Food Health. (2025) 44:261–71. doi: 10.12938/bmfh.2024-127 41050163 PMC12490869

[63] van Gogh M Louwers JM Celli A Gräve S Viveen MC Bosch S . Next-generation IgA-SEQ allows for high-throughput, anaerobic, and metagenomic assessment of IgA-coated bacteria. Microbiome. (2024) 12:211. doi: 10.1186/s40168-024-01923-9 39434178 PMC11492651

[64] DuPont HL Jiang ZD Alexander AS DuPont AW Brown EL . Intestinal IgA-coated bacteria in healthy- and altered-microbiomes (dysbiosis) and predictive value in successful fecal microbiota transplantation. Microorganisms. (2022) 11:93. doi: 10.3390/microorganisms11010093 36677385 PMC9862469

[65] Tabassum N Nakayama-Imaohji H Munyeshyaka E Tada A Kondo T Kondo S . Reactivity of autologous serum IgG to gut microbes in pediatric ulcerative colitis. Int J Mol Sci. (2025) 26:8196. doi: 10.3390/ijms26178196 40943123 PMC12428623

[66] Yang QH Zhang CN . Comparative study on the pathogenesis of Crohn's disease and ulcerative colitis. World J Gastroenterol. (2025) 31:106406. doi: 10.3748/wjg.v31.i19.106406 40497094 PMC12146918

[67] Qin Z Wang R Zhang Y . Dual roles of complement in ulcerative colitis: Insights from clinical studies and animal research. Eur J Med Res. (2025) 30:1234. doi: 10.1186/s40001-025-03400-x 41388435 PMC12699900

[68] Ma Y Zhang K Wu Y Fu X Liang S Peng M . Revisiting the relationship between complement and ulcerative colitis. Scand J Immunol. (2023) 98:e13329. doi: 10.1111/sji.13329 38441324

[69] Kang DY Park JL Yeo MK Kang SB Kim JM Kim JS . Diagnosis of Crohn's disease and ulcerative colitis using the microbiome. BMC Microbiol. (2023) 23:336. doi: 10.1186/s12866-023-03084-5 37951857 PMC10640746

[70] Ito T Kayama H . Roles of fibroblasts in the pathogenesis of inflammatory bowel diseases and IBD-associated fibrosis. Int Immunol. (2025) 37:377–92. doi: 10.1093/intimm/dxaf015 40110813

[71] Eriksen C Moll JM Myers PN Pinto ARA Danneskiold-Samsøe NB Dehli RI . IgG and IgM cooperate in coating of intestinal bacteria in IgA deficiency. Nat Commun. (2023) 14:8124. doi: 10.1038/s41467-023-44007-2 38065985 PMC10709418

[72] Takahashi K Morita N Tamano R Gao P Iida N Andoh A . Mouse IgA modulates human gut microbiota with inflammatory bowel disease patients. J Gastroenterol. (2024) 59:812–24. doi: 10.1007/s00535-024-02121-y 38874761 PMC11339086

[73] Kuijper LH Kreher C Elias G Claireaux M Kerster G Bos AV . Longevity of antibody responses is associated with distinct antigen-specific B cell subsets early after infection. Front Immunol. (2024) 15:1505719. doi: 10.3389/fimmu.2024.1505719 39742271 PMC11686410

[74] Rudbaek JJ Agrawal M Torres J Mehandru S Colombel JF Jess T . Deciphering the different phases of preclinical inflammatory bowel disease. Nat Rev Gastroenterol Hepatol. (2024) 21:86–100. doi: 10.1038/s41575-023-00854-4 37950021 PMC11148654

[75] Huang C Zhu W Li Q Lei Y Chen X Liu S . Antibody Fc-receptor FcϵR1γ stabilizes cell surface receptors in group 3 innate lymphoid cells and promotes anti-infection immunity. Nat Commun. (2024) 15:5981. doi: 10.1038/s41467-024-50266-4 39013884 PMC11252441

[76] Hu C Liao S Lv L Li C Mei Z . Intestinal immune imbalance is an alarm in the development of IBD. Mediators Inflammation. (2023) 2023:1073984. doi: 10.1155/2023/1073984 37554552 PMC10406561

[77] Dong Y Wang T Wu H . Tertiary lymphoid structures in autoimmune diseases. Front Immunol. (2024) 14:1322035. doi: 10.3389/fimmu.2023.1322035 38259436 PMC10800951

[78] Zhong R Guo J Ye W Deng Z Wu H Qi Q . The roles and heterogeneity of CD8+ T cells in inflammatory bowel disease: A narrative review of insights from single-cell transcriptomics (review). Int J Mol Med. (2026) 57:130. doi: 10.3892/ijmm.2026.5801 41860039 PMC13034893

[79] Yamazaki S . Diverse roles of dendritic cell and regulatory T cell crosstalk in controlling health and disease. Int Immunol. (2024) 37:5–14. doi: 10.1093/intimm/dxae042 38953561

[80] Olatunde AC Hale JS Lamb TJ . Cytokine-skewed Tfh cells: Functional consequences for B cell help. Trends Immunol. (2021) 42:536–50. doi: 10.1016/j.it.2021.04.006 33972167 PMC9107098

[81] Rincon-Arevalo H Stefanski AL Le TA Cases M Wiedemann A Szelinski F . Differential response of IgM and IgG memory B cell populations to CD40L: Insights of T cell - memory B cell interactions. Front Immunol. (2024) 15:1432045. doi: 10.3389/fimmu.2024.1432045 39050849 PMC11266000

[82] Humphreys DT Lewis A Pan-Castillo B Berti G Mein C Wozniak E . Single cell sequencing data identify distinct B cell and fibroblast populations in stricturing Crohn's disease. J Cell Mol Med. (2024) 28:e18344. doi: 10.1111/jcmm.18344 38685679 PMC11058334

[83] Pesesky M Bharanikumar R Le Bourhis L ElAbd H Rosati E Carty CL . Antigen-driven expansion of public clonal T-cell populations in inflammatory bowel diseases. J Crohns Colitis. (2025) 19:jjaf048. doi: 10.1093/ecco-jcc/jjaf048 40121186

[84] Melcher C Schramm CA Kampe L Zhang Y Westphal K Krautkrämer M . Inflammatory modalities shape the IgA repertoire via stochastic processes. Cell Rep. (2025) 44:116307. doi: 10.1016/j.celrep.2025.116307 40966088

[85] Simons B Nguyen HT Panyot A Hager FT Kabbert J Laouina A . Clonal persistence dominates homeostatic intestinal IgA responses. Immunity. (2025) 58:3061–3074.e5. doi: 10.1016/j.immuni.2025.11.005 41330367

[86] Cohen RH Colgan SP . Mucosal responses to type II interferon in IBD. Inflammation Bowel Dis. (2025) 31:2584–92. doi: 10.1093/ibd/izaf143 40682561 PMC12455598

[87] Liu HY Yuan P Li S Ogamune KJ Shi X Zhu C . Lactobacillus johnsonii alleviates experimental colitis by restoring intestinal barrier function and reducing NET-mediated gut-liver inflammation. Commun Biol. (2025) 8:1222. doi: 10.1038/s42003-025-08679-4 40813467 PMC12354853

[88] Yang R Shan S Shi J Li H An N Li S . Coprococcus eutactus, a potent probiotic, alleviates colitis via acetate-mediated IgA response and microbiota restoration. J Agric Food Chem. (2023) 71:3273–84. doi: 10.1021/acs.jafc.2c06697 36786768

[89] Cai X Wu W Xu J Lin W Huang P Lin C . Physiologically based pharmacokinetic model of IgG to predict mother-to-fetus transfer of ustekinumab in pregnant patients with inflammatory bowel disease. J Pharm Sci. (2025) 114:103904. doi: 10.1016/j.xphs.2025.103904 40639462

[90] Chen C Xu J Han T Chen G Yu K Du C . Microencapsulation as a protective strategy for sialylated immunoglobulin G: Efficacy in alleviating symptoms of dextran sulfate sodium-induced colitis in mice and potential mechanisms. J Agric Food Chem. (2024) 72:4074–88. doi: 10.1021/acs.jafc.3c07733 38323407

[91] Zhang Z Xu Q Huang L . B cell depletion therapies in autoimmune diseases: Monoclonal antibodies or chimeric antigen receptor-based therapy? Front Immunol. (2023) 14:1126421. doi: 10.3389/fimmu.2023.1126421 36855629 PMC9968396

[92] Kotagiri P Mescia F Rae WM Bergamaschi L Tuong ZK Turner L . B cell receptor repertoire kinetics after SARS-CoV-2 infection and vaccination. Cell Rep. (2022) 38:110393. doi: 10.1016/j.celrep.2022.110393 35143756 PMC8801326

[93] Alemán OR Rosales C . Human neutrophil Fc gamma receptors: Different buttons for different responses. J Leukoc Biol. (2023) 114:571–84. doi: 10.1093/jleuko/qiad080 37437115

[94] Xu B Zhou Z Xiao Y Liu Q Xiao T Lv Z . The pleiotropic effect of complement C5a-C5aR1 pathway in diseases: From immune regulation to targeted therapy. Int J Mol Sci. (2025) 26:11693. doi: 10.3390/ijms262311693 41373839 PMC12691729

[95] Kudo T Shimizu T . Mucosal immune systems of pediatric inflammatory bowel disease: A review. Pediatr Int. (2023) 65:e15511. doi: 10.1111/ped.15511 36799518

[96] Haque M Kaminsky L Abdulqadir R Engers J Kovtunov E Rawat M . Lactobacillus acidophilus inhibits the TNF-α-induced increase in intestinal epithelial tight junction permeability via a TLR-2 and PI3K-dependent inhibition of NF-κB activation. Front Immunol. (2024) 15:1348010. doi: 10.3389/fimmu.2024.1348010 39081324 PMC11286488

[97] Zhao C Lin S . PANoptosis in intestinal epithelium: Its significance in inflammatory bowel disease and a potential novel therapeutic target for natural products. Front Immunol. (2025) 15:1507065. doi: 10.3389/fimmu.2024.1507065 39840043 PMC11747037

[98] Haroun C Kroese FGM Verstappen GM . Fc receptor-like proteins and their role in B-cell responses and autoimmune diseases. Immunol Lett. (2026) 279:107144. doi: 10.1016/j.imlet.2026.107144 41617145

[99] Wang Q Feng D Song Y Hu Z Lu Q Zhao M . Biased usage of V/D/J genes and clonal diversity in IgG repertoires correlates with disease activity and clinical features in systemic autoimmune diseases. Immunol Invest. (2025) 54:1461–81. doi: 10.1080/08820139.2025.2550374 40905528

[100] Argyris DG Johnson L Hägglöf T Filippou PS Karagiannis GS . Emerging involvement of CXCL13 in cancer development and progression. Cytokine Growth Factor Rev. (2026) 87:73–88. doi: 10.1016/j.cytogfr.2025.12.005 41389700

[101] Lowry E Chellappa RC Penaranda B Sawant KV Wakamiya M Garofalo RP . CXCL17 is a proinflammatory chemokine and promotes neutrophil trafficking. J Leukoc Biol. (2024) 115:1177–82. doi: 10.1093/jleuko/qiae028 38298146 PMC11135614

[102] Giblin SP Pease JE . What defines a chemokine? - The curious case of CXCL17. Cytokine. (2023) 168:156224. doi: 10.1016/j.cyto.2023.156224 37210967

[103] Fagnano E Pendharkar S Colton M Jones PN Sallan MC Klymenko T . Stromal cell inhibition of anti-CD20 antibody mediated killing of B-cell Malignancies. Front Cell Dev Biol. (2023) 11:1270398. doi: 10.3389/fcell.2023.1270398 38020903 PMC10646167

[104] Stockfelt M Teng YKO Vital EM . Opportunities and limitations of B cell depletion approaches in SLE. Nat Rev Rheumatol. (2025) 21:111–26. doi: 10.1038/s41584-024-01210-9 39815102

[105] Cottignies-Calamarte A Tudor D Bomsel M . Antibody Fc-chimerism and effector functions: When IgG takes advantage of IgA. Front Immunol. (2023) 14:1037033. doi: 10.3389/fimmu.2023.1037033 36817447 PMC9933243

[106] Kato A Toshima A Takekawa G Miyagi H Kubota A Hori S . Immunoglobulin G Fc engineering for localized therapy: Eliminating neonatal Fc receptor interactions and ensuring purification using protein A. Protein Sci. (2025) 34:e70351. doi: 10.1002/pro.70351 41123423 PMC12542301

[107] Meudt M Baumeister J Russell AC Dzielak L Hansen G Mizaikoff B . Glycan pairing in therapeutic IgG orchestrates Fcγ receptor engagement and ADCC: an integrated structure-function approach for thorough evaluation of Fc N-glycans as critical quality attributes. MAbs. (2026) 18:2652642. doi: 10.1080/19420862.2026.2652642 41925250 PMC13048574

[108] Chen Z Wang M Duan W Xia Y Liu H Qian F . Modulating the complement system through epitope-specific inhibition by complement C3 inhibitors. J Biol Chem. (2025) 301:108250. doi: 10.1016/j.jbc.2025.108250 39894217 PMC11910092

[109] Ho BHT Spicer BA Dunstone MA . Action of the terminal complement pathway on cell membranes. J Membr Biol. (2025) 258:269–304. doi: 10.1007/s00232-025-00343-6 40122920 PMC12313776

[110] Jia C Yang X Zhao MH Tan Y Xiao J . Complement C3 recognition by C3 convertases. Sci Adv. (2026) 12:eadz5404. doi: 10.1126/sciadv.adz5404 41719406 PMC12922740

[111] Elesela S Arzola-Martínez L Rasky A Ptaschinski C Hogan SP Lukacs NW . Mucosal IgA immune complex induces immunomodulatory responses in allergic airway and intestinal TH2 disease. J Allergy Clin Immunol. (2023) 152:1607–1618.e1. doi: 10.1016/j.jaci.2023.08.006 37604310

[112] Gao P Morita N Shinkura R . Role of mucosal IgA antibodies as novel therapies to enhance mucosal barriers. Semin Immunopathol. (2024) 47:1. doi: 10.1007/s00281-024-01027-4 39567378 PMC11579142

[113] De Ponte Conti B Marino R Rezzonico-Jost T Forcato M Mangani D Notario E . Secretory IgA amplification during immune checkpoint blockade enhances the control of tumor growth by enterotropic T cells. Sci Adv. (2025) 11:eaeb5308. doi: 10.1126/sciadv.aeb5308 41042869 PMC12494019

[114] Boucher A Anderson C Hinman R Kindschuh M Fung J Wang T . Engineered human B cells targeting tumor-associated antigens exhibit antigen presentation and antibody-mediated functions. Front Immunol. (2025) 16:1621222. doi: 10.3389/fimmu.2025.1621222 40808959 PMC12343648

[115] Pitner RA Chao JL Dahl NP Fan MN Cai X Avery NG . Blunting specific T-dependent antibody responses with engineered "decoy" B cells. Mol Ther. (2024) 32:3453–69. doi: 10.1016/j.ymthe.2024.08.023 39192583 PMC11489556

[116] Kumric M Zivkovic PM Ticinovic Kurir T Vrdoljak J Vilovic M Martinovic D . Role of B-cell activating factor (BAFF) in inflammatory bowel disease. Diagnostics (Basel). (2021) 12:45. doi: 10.3390/diagnostics12010045 35054212 PMC8774757

[117] Jha D Mehandru S . B cell dysregulation in IBD: From pathophysiology to therapeutic targeting. Inflammation Bowel Dis. (2026) 32:830–3. doi: 10.1093/ibd/izaf311 41500502

[118] Betzler AC Ushmorov A Brunner C . The transcriptional program during germinal center reaction - a close view at GC B cells, Tfh cells and Tfr cells. Front Immunol. (2023) 14:1125503. doi: 10.3389/fimmu.2023.1125503 36817488 PMC9936310

[119] Wang AA Luessi F Neziraj T Pössnecker E Zuo M Engel S . B cell depletion with anti-CD20 promotes neuroprotection in a BAFF-dependent manner in mice and humans. Sci Transl Med. (2024) 16:eadi0295. doi: 10.1126/scitranslmed.adi0295 38446903

[120] Carreto-Binaghi LE Sztein MB Booth JS . Role of cellular effectors in the induction and maintenance of IgA responses leading to protective immunity against enteric bacterial pathogens. Front Immunol. (2024) 15:1446072. doi: 10.3389/fimmu.2024.1446072 39324143 PMC11422102

[121] Neu SD Gurski CJ Meinhardt NJ Jennings KC Dittel BN . Gut IgA-antibody secreting cells segregate into four Blimp1+ subsets based on differential expression of IgA and Ki-67 and are retained following prolonged αCD20 B cell depletion in mice. J Immunol. (2025) 214:780–94. doi: 10.1093/jimmun/vkae046 40073093 PMC12041773

[122] Beneduce C Nguyen S Washburn N Schaeck J Meccariello R Holte K . Inhibitory Fc-gamma IIb receptor signaling induced by multivalent IgG-Fc is dependent on sialylation. Cells. (2023) 12:2130. doi: 10.3390/cells12172130 37681862 PMC10486564

[123] Frischauf N Strasser J Borg EGF Labrijn AF Beurskens FJ Preiner J . Complement activation by IgG subclasses is governed by their ability to oligomerize upon antigen binding. Proc Natl Acad Sci USA. (2024) 121:e2406192121. doi: 10.1073/pnas.2406192121 39436656 PMC11536094

[124] Damelang T de Taeye SW Rentenaar R Roya-Kouchaki K de Boer E Derksen NIL . The influence of human IgG subclass and allotype on complement activation. J Immunol. (2023) 211:1725–35. doi: 10.4049/jimmunol.2300307 37843500 PMC10656437

[125] Dobó J Kocsis A Farkas B Demeter F Cervenak L Gál P . The lectin pathway of the complement system-activation, regulation, disease connections and interplay with other (proteolytic) systems. Int J Mol Sci. (2024) 25:1566. doi: 10.3390/ijms25031566 38338844 PMC10855846

[126] Ko H Kim CJ Choi S Noh J Kim SW Lee J . Commensal microbe-derived butyrate enhances T follicular helper cell function to boost mucosal vaccine efficacy. Microbiome. (2026) 14:37. doi: 10.1186/s40168-025-02284-7 41566359 PMC12825270

[127] Zeng S Wang S Mu D . Metagenomics for IgA-coated gut microbiota: from taxonomy to function. Trends Microbiol. (2025) 33:823–5. doi: 10.1016/j.tim.2025.04.001 40246602

[128] Shimizu A Tsuboi N Hatanaka S Fukunaga S Sasaki T Haruhara K . Distinct clinicopathological features of IgA nephropathy associated with Crohn's disease: comparison with ulcerative colitis and non-IBD IgA nephropathy. Clin Exp Nephrol. 30:757–66. doi: 10.1007/s10157-026-02850-9 41870749

[129] Khedkar S Khan MA . An *in vitro* study elucidating the synergistic effects of aqueous cinnamon extract and an anti-TNF-α biotherapeutic: implications for a complementary and alternative therapy for non-responders. BMC Complement Med Ther. (2024) 24:131. doi: 10.1186/s12906-024-04438-w 38521924 PMC10960381

[130] Szaflarska A Lenart M Rutkowska-Zapała M Siedlar M . Clinical and experimental treatment of primary humoral immunodeficiencies. Clin Exp Immunol. (2024) 216:120–31. doi: 10.1093/cei/uxae008 38306460 PMC11036112

[131] Cramer P Neys SFH Fiedler M Lorenzetti R Reinhard H Janowska I . A viral glycoprotein targets IgG+ memory B cells to mediate humoral immune evasion. EMBO Mol Med. (2026) 18:795–823. doi: 10.1038/s44321-026-00372-1 41559372 PMC12905349

[132] Alashkar Alhamwe B Yuskaeva K Wulf F Trinkmann F Kriegsmann M Thomas M . Peripheral inflammation featuring eosinophilia or neutrophilia is associated with the survival and infiltration of eosinophils within the tumor among various histological subgroups of patients with NSCLC. Int J Mol Sci. (2024) 25:9552. doi: 10.3390/ijms25179552 39273499 PMC11395097

[133] Bagheri Y Moeini Shad T Namazi S Tofighi Zavareh F Azizi G Salami F . B cells and T cells abnormalities in patients with selective IgA deficiency. Allergy Asthma Clin Immunol. (2023) 19:23. doi: 10.1186/s13223-023-00775-6 36941677 PMC10029301

[134] Zhang H Xiang J Feng J Zhang M Xi Q . Gut microbiome dysbiosis and inflammatory bowel disease complement each other. Dig Dis. (2025) 43:345–57. doi: 10.1159/000544771 39999800

[135] He L Li X Jiang S Ou Y Wang S Shi N . The influence of the gut microbiota on B cells in autoimmune diseases. Mol Med. (2025) 31:149. doi: 10.1186/s10020-025-01195-5 40264032 PMC12016346

[136] Park YE Park SB Kang SB Park Y Kim DS Na SY . Long-term clinical course, treatment patterns, and prognosis in pediatric-onset vs. adult-onset IBD: a multicenter retrospective cohort study in Korea. BMC Gastroenterol. (2026) 26:125. doi: 10.1186/s12876-026-04630-x 41566234 PMC12905910

[137] Helmin-Basa A Kopoń M Koza J Strzyżewska E Skalska-Bugała A Leśniewski F . Distinct B cell subsets changes as potential biomarkers of response to biologic therapy in Crohn's disease. Int J Mol Sci. (2025) 26:9539. doi: 10.3390/ijms26199539 41096805 PMC12525053

[138] Neurath MF Sands BE Rieder F . Cellular immunotherapies and immune cell depleting therapies in inflammatory bowel diseases: the next magic bullet? Gut. (2024) 74:9–14. doi: 10.1136/gutjnl-2024-332919 39025492 PMC11671923

[139] Renga G Nunzi E Stincardini C Pariano M Puccetti M Pieraccini G . CPX-351 exploits the gut microbiota to promote mucosal barrier function, colonization resistance, and immune homeostasis. Blood. (2024) 143:1628–45. doi: 10.1182/blood.2023021380 38227935

[140] Wang Z Zhen C Guo X Qu M Zhang C Song J . Landscape of gut mucosal immune cells showed gap of follicular or memory B cells into plasma cells in immunological non-responders. Clin Transl Med. (2024) 14:e1699. doi: 10.1002/ctm2.1699 38783408 PMC11116468

[141] Yang Q Zhang F Chen H Hu Y Yang N Yang W . The differentiation courses of the Tfh cells: a new perspective on autoimmune disease pathogenesis and treatment. Biosci Rep. (2024) 44:BSR20231723. doi: 10.1042/BSR20231723 38051200 PMC10830446

[142] Bouteau A Qin Z Zurawski S Zurawski G Igyártó BZ . Langerhans cells drive Tfh and B cell responses independent of canonical cytokine signals. bioRxiv. (2025), 2025.01.10.632426. doi: 10.1101/2025.01.10.632426. Preprint. 40755758 PMC12315773

[143] Mahdy AKH Schöpfel V Huppertz-Hauss G Perminow G Tran F Bang C . Simultaneous profiling of the blood and gut T and B cell repertoires in Crohn's disease and symptomatic controls illustrates tissue-specific alterations in the immune repertoire of individuals with Crohn's disease. Front Immunol. (2025) 16:1638522. doi: 10.3389/fimmu.2025.1638522 40977727 PMC12447526

[144] Collet A Guerrier T Sanges S Chépy A Sobanski V Launay D . Autoreactive B cells in autoimmune diseases: Mechanisms, functions and clinical implications. Autoimmun Rev. (2025) 24:103851. doi: 10.1016/j.autrev.2025.103851 40494440

[145] Sarrand J Soyfoo M . B-cells and plasmablasts as architects of autoimmune disease: From molecular footprints to precision therapeutics. Cells. (2026) 15:119. doi: 10.3390/cells15020119 41597194 PMC12839912

[146] Kocabiyik O Amlashi P Vo AL Suh H Rodriguez-Aponte SA Dalvie NC . Vaccine targeting to mucosal lymphoid tissues promotes humoral immunity in the gastrointestinal tract. Sci Adv. (2024) 10:eadn7786. doi: 10.1126/sciadv.adn7786 38809992 PMC11135404

[147] Jones AT Marino AE Martynyuk T Bournazos S Ravetch JV . The anti-inflammatory activity of IgG is enhanced by co-engagement of type I and II Fc receptors. Science. (2025) 390:eadv2927. doi: 10.1126/science.adv2927 41197001 PMC13426471

[148] Sneed SL Strandberg EA Laureano AFS Vattepu R Sun Y Tran TT . Molecular determinants of sialylated IgG anti-inflammatory activity. Proc Natl Acad Sci USA. (2025) 122:e2411600122. doi: 10.1073/pnas.2411600122 40377989 PMC12107084

[149] Conrey PE Denu L O'Boyle KC Rozich I Green J Maslanka J . IgA deficiency destabilizes homeostasis toward intestinal microbes and increases systemic immune dysregulation. Sci Immunol. (2023) 8:eade2335. doi: 10.1126/sciimmunol.ade2335 37235682 PMC11623094

[150] Yang C Chen-Liaw A Spindler MP Tortorella D Moran TM Cerutti A . Immunoglobulin A antibody composition is sculpted to bind the self gut microbiome. Sci Immunol. (2022) 7:eabg3208. doi: 10.1126/sciimmunol.abg3208 35857580 PMC9421563

[151] Mestrovic A Perkovic N Bozic D Kumric M Vilovic M Bozic J . Precision medicine in inflammatory bowel disease: A spotlight on emerging molecular biomarkers. Biomedicines. (2024) 12:1520. doi: 10.3390/biomedicines12071520 39062093 PMC11274502

[152] Pelissier A Luo S Stratigopoulou M Guikema JEJ Rodríguez Martínez M . Exploring the impact of clonal definition on B-cell diversity: Implications for the analysis of immune repertoires. Front Immunol. (2023) 14:1123968. doi: 10.3389/fimmu.2023.1123968 37138881 PMC10150052

[153] Wang Y Li R Tong R Chen T Sun M Luo L . Integrating single-cell RNA and T cell/B cell receptor sequencing with mass cytometry reveals dynamic trajectories of human peripheral immune cells from birth to old age. Nat Immunol. (2025) 26:308–22. doi: 10.1038/s41590-024-02059-6 39881000 PMC11785523

[154] Baars MJD Floor E Sinha N Ter Linde JJM van Dam S Amini M . Multiplex spatial omics reveals changes in immune-epithelial crosstalk during inflammation and dysplasia development in chronic IBD patients. iScience. (2024) 27:110550. doi: 10.1016/j.isci.2024.110550 39165839 PMC11334790

